# The role of peripheral nerve fibers and their neurotransmitters in cartilage and bone physiology and pathophysiology

**DOI:** 10.1186/s13075-014-0485-1

**Published:** 2014-11-22

**Authors:** Susanne Grässel

**Affiliations:** Department of Orthopaedic Surgery, Experimental Orthopaedics, University of Regensburg, ZMB/BioPark 1, Josef-Engert-Str. 9, 93053 Regensburg, Germany

## Abstract

The peripheral nervous system is critically involved in bone metabolism, osteogenesis, and bone remodeling. Nerve fibers of sympathetic and sensory origin innervate synovial tissue and subchondral bone of diathrodial joints. They modulate vascularization and matrix differentiation during endochondral ossification in embryonic limb development, indicating a distinct role in skeletal growth and limb regeneration processes. In pathophysiological situations, the innervation pattern of sympathetic and sensory nerve fibers is altered in adult joint tissues and bone. Various resident cell types of the musculoskeletal system express receptors for sensory and sympathetic neurotransmitters. Osteoblasts, osteoclasts, mesenchymal stem cells, synovial fibroblasts, and different types of chondrocytes produce distinct subtypes of adrenoceptors, receptors for vasointestinal peptide, for substance P and calcitonin gene-related peptide. Many of these cells even synthesize neuropeptides such as substance P and calcitonin gene-related peptide and are positive for tyrosine-hydroxylase, the rate-limiting enzyme for biosynthesis of catecholamines. Sensory and sympathetic neurotransmitters modulate osteo-chondrogenic differentiation of mesenchymal progenitor cells during endochondral ossification in limb development. In adults, sensory and sympathetic neurotransmitters are critical for bone regeneration after fracture and are involved in the pathology of inflammatory diseases as rheumatoid arthritis which manifests mainly in joints. Possibly, they might also play a role in pathogenesis of degenerative joint disorders, such as osteoarthritis. All together, accumulating data imply that sensory and sympathetic neurotransmitters have crucial trophic effects which are critical for proper limb formation during embryonic skeletal growth. In adults, they modulate bone regeneration, bone remodeling, and articular cartilage homeostasis in addition to their classic neurological actions.

## Introduction

Clinical observations demonstrated the importance of the peripheral nervous system for maintaining body homeostasis and mediating organogenesis and tissue repair. It is reported that in patients with head injuries, fractures frequently heal with excessive callus formation and at a faster rate than normal [1]. In patients with spinal cord injuries, a profound decrease of sublesional bone mineral density was measured in comparison with controls [2]. This loss of bone mass is higher at the distal femur and proximal tibia, which are the most common sites of fractures. In addition, hip fracture after stroke is an increasingly recognized problem. Measures to prevent bone loss, which is a potent risk factor for fracture, and to preserve bone architecture have not been part of stroke management thus far [3].

These and other recent publications suggest that the peripheral nervous system is critically involved in bone metabolism, osteogenic differentiation of precursor cells (osteogenesis), bone mineralization, and bone remodeling [4,5]. Nerve fibers of sympathetic and sensory origin frequently innervate trabecular bone, periosteum, and fracture callus [6,7]. They are involved in controlling vascularization and matrix differentiation during endochondral ossification in embryonic limb development [8], indicating a distinct role in modulating skeletal growth and limb formation processes.

On one hand, disorders of nerves (central or peripheral) can have substantial influence on bone health, repair, and regeneration. On the other hand, dramatic alterations in density and distribution of sympathetic and sensory nerve fibers are reported in musculoskeletal pathophysiology. Changes in the density of sympathetic nerve fibers, which are characterized by tyrosine-hydroxylase (TH) or neuropeptide Y (NPY) or both, in synovial tissue contribute to rheumatoid arthritis (RA) [9]. Loss of sensory joint innervation during aging is suggested to accelerate degenerative cartilage alterations which contribute to development of spontaneous osteoarthritis (OA) in mice [10]. Capsaicin-sensitive sensory neurons contribute to the maintenance of tibial and femoral metaphyseal trabecular bone integrity and bone mass [11], suggesting a positive role in bone regeneration, whereas with respect to the sympathetic nervous system, some studies reported a favorable influence of β-blockers on bone mass and reducing fracture risk [12].

Sensory nerves in general contain two different nociceptive neuropeptide families: the tachykinins [13] and calcitonin gene-related peptides (CGRPs). Effects of all tachykinins are mediated by three neurokinin receptors with varying affinities for the individual ligands [14] (Table 1). Classically, tachykinin substance P (SP) is known as a mediator of nociception and of inflammation [15]. CGRP is encoded together with calcitonin and is generated by alternative splicing [16] and signals through a complex family of receptor proteins [17] (Table 1). The most important neurotransmitter of the catecholaminergic sympathetic nervous system is norepinephrine (NE), which signals through α- and β-adrenoceptors (ARs), depending on concentration [18] (Table 2). Vasoactive intestinal peptide (VIP) belongs to a family of structurally related peptides, including secretin, glucagon, gastric inhibitory peptide, growth hormone-releasing factor, and pituitary adenylate cyclase-activating polypeptide (reviewed in [19]). The three different subtypes of VIP receptors belong to the type II family of G protein-coupled receptors (Table 2). Here, a concise overview of efferent functions and roles of sympathetic and sensory nerve fibers and their neurotransmitters in bone and cartilage physiology and pathophysiology is presented.

**Table 1:** **Mammalian sensory neurotransmitters and their receptors**

**Neurotransmitters**	**Receptor (highest affinity)**	**Neuropeptide genes**	**Neuropeptide receptor genes**
Substance P (SP)	Neurokinin (NK)_1_ receptor	Tachykinin (*TAC*) 1 or *PPT-A* or *PPT-I*	Tachykinin receptor (*TACR*) 1
Neurokinin A (NKA)	NK_2_ receptor	*TAC1*	*TACR2*
Alternatively spliced forms are neuropeptides (NP) K and NPγ.			
Neurokinin B (NKB)	NK_3_ receptor	*TAC3* or *PPT-B* or *PPT-II*	*TACR3*
Hemokinin 1 (HK1)	NK_1_ receptor, HK1 receptor?	*TAC4* or *PPT-C*	*TACR1*
Alternatively spliced forms are endokinins (EK) A, B, C, D.			
Alpha-calcitonin-gene-related peptide (αCGRP)	Calcitonin receptor-like receptor (CRLR)/receptor activity-modifying protein (RAMP-1)	*CALCA*	*CALCR/RAMP1*
Beta-calcitonin gene-related peptide (βCGRP)	CRLR/RAMP-1	*CALCB*	*CALCR/RAMP1*

**Table 2:** **Mammalian sympathetic neurotransmitters and their receptors**

**Neurotransmitters**	**Receptors**	**Neuropeptide genes**	**Neurotransmitter receptor genes**
Catecholaminergic			
Norepinephrine or noradrenaline (NA)	≤10^−8^ M: α 1a-, b-, d- adrenoceptors		*ADRA1A, ADRA1B, ADRA1D*
	≤10^−8^ M: α 2a-, b-, c-adrenoceptors		*ADRA2A, ADRA2B, ADRA2C*
	≥10^−6^ M: β1-, β2-, β3-adrenoceptors		*ADRB1, ADRB2, ADRB3*
Peptidergic			
Vasoactive intestinal peptide (VIP)	VIP-1, VIP-2, and VIP/PACAP receptors	*VIP*	*VIPR1, VIPR2*

PACAP, pituitary adenylate cyclase-activating polypeptide.

## Sensory and sympathetic nerve fibers in cartilage physiology and development

**Figure 1:**
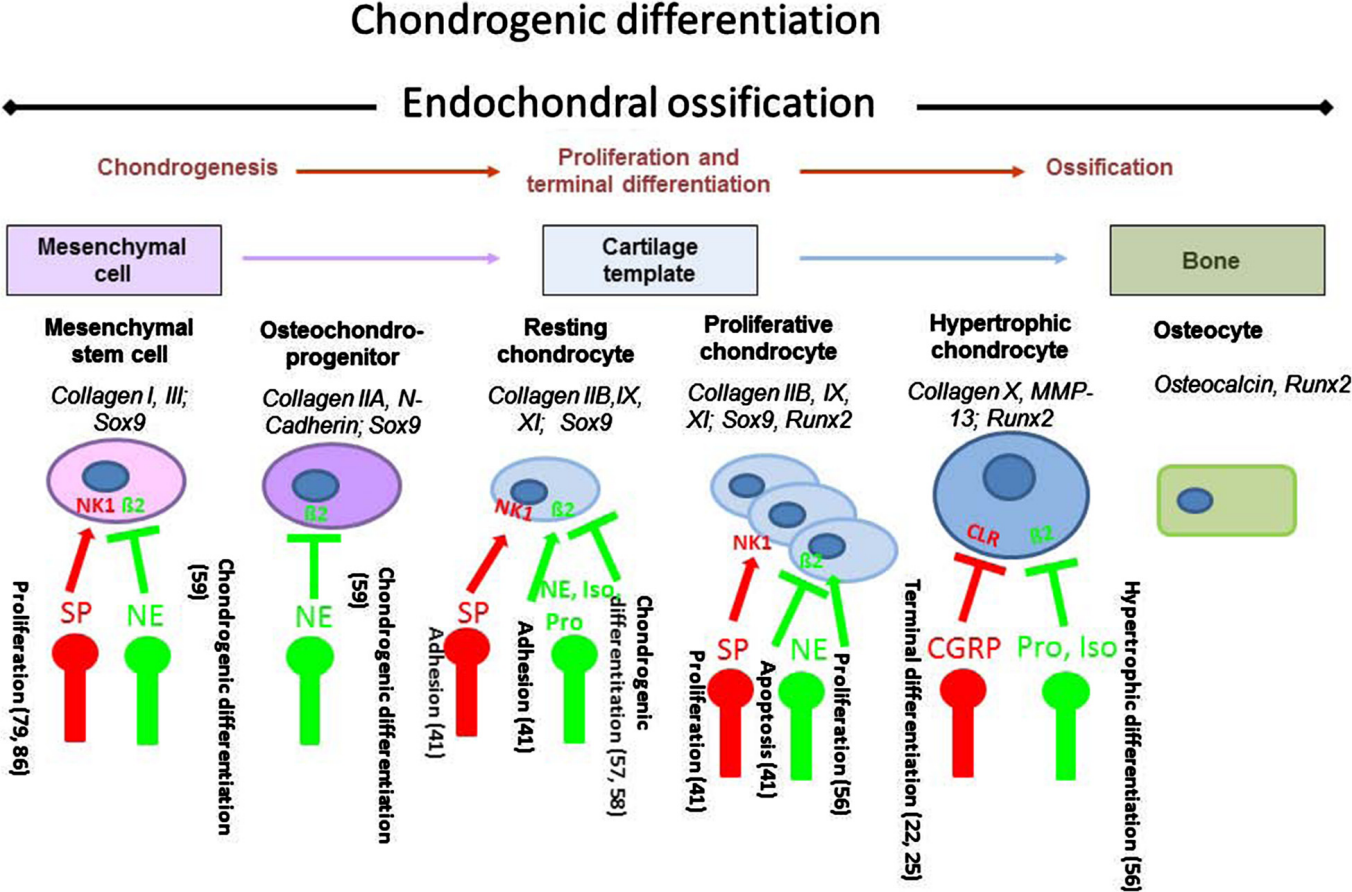
**Role of sensory and sympathetic neurotransmitters and their receptors in chondrogenic differentiation.** Sensory - substance P (SP) and calcitonin gene-related peptide (CGRP) - and sympathetic (norepinephrine; NE) neurotransmitters and antagonists/agonists (isoproterenol and propranolol) affect chondrogenic differentiation and metabolism of chondroprogenitor cells and bone marrow-derived stem cells (BMSCs). These neurotropic effects are mediated through specific receptors for sensory neuropeptides, neurokinin 1 (NK1) receptor and CGRP receptor (CLR) and mainly the sympathetic β2-adrenoceptor. A line with an arrow indicates stimulation, and a line with a bar indicates inhibition. The red (green) nerve ending represent sensory (sympathetic) nerve fibers. Numbers indicate references according to bibliography at the end of this review. β2, β2-adrenoceptor; Iso, isoproterenol; Pro, propranolol.

In diarthrodial joints, permanent hyaline cartilage covers the surface of bones and enables them to bear very large compressive loads without distortion and allows smooth, frictionless movement of the joints [20]. Importantly, unlike other musculoskeletal connective tissues such as periosteum and synovium, cartilage does not contain blood vessels and is not deeply innervated by nerve fibers, indicating that cartilage for some reason might be a hostile environment for the spreading of nerve fibers. However, there is sparse evidence that sensory nerve fibers come into contact with those chondrocytes located in growth plate cartilage and in the outer layer of articular cartilage. In rat knee joints, CGRP-positive fibers which originate from periosteum and near insertion regions of muscle and tendons penetrate the outer layer of articular and meniscus cartilage up to 25 μm and are located between single chondrocytes, indicating a local effector function. However, there are subpopulations of SP-positive axons in perichondrium and periosteum which for unknown reasons do not innervate the cartilage [21]. CGRP- and SP-positive nerve fibers precede the development of cartilage canals which are formed during skeletal growth shortly after birth and were detected when they penetrated the canals of growth cartilage in the epiphysis of young rats, thereby coming into close contact with chondrocytes [22,23]. The development of the secondary ossification center is subsequent to the presence of cartilage canals carrying sensory nerve fibers, and it is speculated that these nerve fibers modulate the formation of synovial joints through trophic effects [24]. This observation implies important functions of sensory nerves for regulating chondrogenic differentiation during limb growth in embryonic development. In line with this, Edoff and colleagues [22,25] reported that articular chondrocytes respond specifically to the application of CGRP by increasing the cAMP level. They assume that dorsal root ganglion neurons which project to growth plate cartilage may influence chondrocyte differentiation via CGRP. It is described that increased levels of cAMP suppress terminal differentiation of chondrocytes and matrix mineralization [26] which makes it likely that local release of CGRP can delay chondrocyte hypertrophy and subsequent terminal differentiation through modulating cAMP level (Figure 1). Whereas sympathetic nerve fibers have been abundantly localized to subchondral bone marrow [27] and synovial joint tissues [28], no reports exist about close contact to chondrocytes or innervation of healthy articular cartilage.

## Sensory and sympathetic nerve fibers in osteoarthritis

OA, a degenerative disorder of diathrodial joints culminating in the irreversible destruction of the articular cartilage, is clinical highly relevant and a burden for the health-care system and society because of high costs for diagnosis, treatment, sick leave, rehabilitation, and early retirement [29]. However, OA is not exclusively a disorder of articular cartilage; it can be considered an organ failure of the whole joint with additional abnormalities especially in subchondral bone, ligaments, synovium, and the joint capsule. Suri and colleagues [30] have localized both sensory (SP- and CGRP-positive) and sympathetic (NPY-positive) nerve fibers in similar distributions to the articular cartilage in human tibiofemoral OA. In the pathogenesis of OA, nerves grow into joint structures through vascular channels mainly from subchondral bone breaching through the tidemark. The exclusively perivascular localization of nerves in the articular cartilage implies that vascularization is the driving force behind its innervation. Notably, vascularization of the non-calcified cartilage was found throughout a wide range of histological OA stages and was not restricted to end-stage OA. Possibly, scoring of nervous innervation and vascularization of cartilage might be exploited as a measure for severity of degradative changes in OA pathogenesis. The authors suggest that innervation of the articular cartilage is therefore a potential source of pain in patients with knee OA. These authors and others have also shown sympathetic and perivascular sensory innervation of tibial osteophytes, the latter localized to the base of osteophytes [30,31]. Sensory innervation of osteophytes may explain why radiological grading of osteophytosis is associated with reported pain severity [32]. However, despite the clinical importance of pain in OA, pain mechanisms are poorly understood. It is unclear which structures in the joint give rise to OA pain, and the nature of OA pain (nociceptive versus neuropathic) is a matter of ongoing discussion [33].

In line with Suri and colleagues [30], it was recently demonstrated that there was a marked preponderance of sensory over sympathetic nerve fibers in ankle bone and synovial tissue in patients with OA compared with patients with anterior knee pain after primary arthroplasty [34]. In patients with chronic RA and end-stage OA, this study of synovial tissues demonstrated a preponderance of SP-positive nerve fibers relative to CGRP-positive nerve fibers in patients with RA but not in patients with end-stage OA. Here, an even balance exists between SP-positive and CGRP-positive nerve fibers. Finally, the authors observed a positive correlation between the density of CGRP-positive and sympathetic nerve fibers in OA, which they interpreted as an anti-inflammatory signal. Such a positive correlation was not observed in synovial tissue of patients with RA, which demonstrates a markedly reduced presence of sympathetic nerve fibers. Thus, in the less-inflamed tissue of patients with OA, coupling of two anti-inflammatory pathways might dampen the inflammatory process, a situation that is lost in the tissue of patients with RA, indicating a neuroendocrine uncoupling mechanism [9]. A drastic loss of specifically sympathetic nerve fibers in synovial joint tissues of patients with RA has previously been demonstrated [35]; however, it appears that during OA pathogenesis this does not happen. Instead, inflammatory changes in synovium of OA joints are associated with a massive destruction of the total capillary and neuronal, sympathetic and sensory nervous network which is present in normal synovium [36]. Notably, in a murine OA model, it was suggested that in some soft joint tissues, CGRP- and SP-positive nerve fibers disappeared 5 weeks after induction of OA with intra-articular injection of collagenase [37]. This quite aggressive method of OA induction leads to profound degenerative alterations in joint tissue after a few weeks, comparable with late-stage OA in humans. The mechanisms which destruct the normal vascular and neural network are not identified yet, and also it is unclear whether nerves are destroyed as a consequence of OA or whether pathogenesis of OA is facilitated because of nerve disappearance. A study by Salo and colleagues [10] indicated that loss of SP- and CGRP-positive joint innervation always preceded histological changes of cartilage degeneration. They used a mice model which usually developed a mild form of OA, but surgical ablation of joint innervation caused the development of severe patellofemoral OA. Their findings would be consistent with the hypothesis that an age-related loss of joint innervation may contribute to the development of OA. Whether this is comparable to the human situation remains to be investigated, and insight into these mechanisms will require reproducible OA animal models resembling slow proceeding pathogenesis of human OA and enabling longitudinal studies from early onset of the disease on to late stages.

## Nerve repellent factors in cartilage

Why it is that healthy and also mostly diseased articular cartilage is basically not innervated is still not sufficiently clarified. Possibly, the lack of blood vessels prevents innervation or vice versa. Trying to find underlying molecular mechanisms for this unique phenomenon suggests the presence of specific axon guidance or nerve repellent factors in cartilage. Well known sensory and sympathetic nerve-specific axon guidance molecules or repellent factors belong to the semaphorin family. A selective repellent of SP- and CGRP-positive sensory nerve fibers is semaphorin (Sema)3A, which, besides being expressed in neuronal tissues, is expressed in developing cartilage and bone [38], in intervertebral discs [39] and recently was even found in adult articular cartilage [40]. Gomez and colleagues [38] showed convincingly that the Sema signaling system consisting of Sema3A and its receptors plexin (Plx)-A3/neuropilin (NP)-1 is expressed in resting, pre-hypertrophic, and hypertrophic chondrocytes in growth cartilage before the onset of neurovascular invasion during endochondral ossification. They suggest that the Sema3A/Plx-A3/NP-1 pathway would inhibit neuro-vascularization of the cartilage anlage early in skeletal development. This block could be removed later and locally by the downregulation of key receptor chains, the elimination of Sema3A-producing cells (for example, hypertrophic chondrocytes), or by the expression of competitors of Sema3A (such as vascular endothelial growth factor (VEGF)_165_), for instance, by perichondral cells and hypertrophic chondrocytes. Inhibition of Sema3A signaling would then allow blood vessel and nerve fiber invasion of the diaphysis and thus the onset of endochondral ossification together with the start of local neural or vascular regulation (or both) of bone formation. Later, during the ossification process, Sema3A may provide a repulsive guidance, allowing the growing nerve fibers to be directed to their targets in bone tissue. Another group reported that gene and protein expression of Sema3A and its receptor NP-1 was significantly elevated in chondrocytes from OA cartilage as compared with chondrocytes from normal cartilage and that the Sema3A expression is closely correlated with chondrocyte cloning, which is a characteristic feature of OA cartilage [40]. The authors imply the possibility that Sema3A plays a role in the pathogenesis of chondrocyte cloning through antagonizing and inhibition of VEGF-mediated cell migration. All together, these studies assign a novel function to Sema in chondrogenic differentiation during embryonic development and cartilage degeneration in adults in addition to their known axon guidance role.

## Sensory and sympathetic neurotransmitters and their receptors expressed by chondrocytes

Besides their classic function in nociception, SP and CGRP appear to have extra functions in the musculoskeletal system. Lately, murine costal and human articular chondrocytes have been recognized to endogenously produce SP and its receptor NK1 [41,42]. Earlier, SP was immunolocalized to articular cartilage of dog shoulder joints. Expression and localization were increased in chondrocytes and within the extracellular matrix after low-impact regimented exercise, indicating a role in signaling pathways through which chondrocytes respond to mechanical stimulation [43]. This was indeed demonstrated by Millward-Sadler and colleagues [44], who suggested that SP is involved in mechanotransduction via the NK1 receptor. They found SP to be necessary for a hyperpolarization response of the cell membrane, and concomitant changes in gene expression as a response to mechanical stimulation indicate a role of SP in the maintenance of articular cartilage matrix integrity and function. The same group demonstrated that normal and OA chondrocytes reacted differently to mechanical stimulation in that OA chondrocytes upregulated gene expression of the SP encoding gene, *Tac1*, whereas non-OA chondrocytes did not respond with changes in *Tac1* gene expression [45]. In addition, we recently demonstrated that costal chondrocytes from newborn mice when stimulated with SP induced proliferation and cell-matrix adherence by stimulating formation of focal adhesion contacts. These effects are mediated specifically through the NK1 receptor [41]. Our observation implies that SP might modulate proliferation rate of growth plate chondrocytes and consequently terminal differentiation during endochondral ossification. It is thus conceivable that, in chondrocyte physiology and in chondrogenic differentiation during skeletal growth, endogenous SP production acts as a trophic, anabolic factor and does not function as a classic neuropeptide (Figure 1 and Table 3). However, in adults, the detection of higher levels of SP in synovial fluid from patients with RA and OA, and increased expression of NK1R, indicates catabolic effects of SP on articular cartilage [46]. In addition, transforming growth factor-beta and basic fibroblast growth factor play an important role as inductor or promoter for production of SP in synovial fibroblasts. These data are supported by Im and colleagues [47], who elegantly demonstrated that SP induces interleukin 1-beta (IL-1β) release. They propose a mechanism by which basic fibroblast growth factor, together with SP, reduces proteoglycan deposition and stimulates production and release of matrix metalloprotease (MMP)-13 in human articular chondrocytes and thus accelerates catabolic processes in cartilage. All together, these observations suggest that SP has autocrine functions and can modulate chondrocyte metabolism and cartilage homeostasis differentially during skeletal growth and in pathophysiology. As in synovial cells where SP is described as potent mediator of inflammation by promoting secretion of prostaglandin E2, several MMPs [48], reactive oxygen species [49], IL-1, and tumor necrosis factor-alpha [50], SP also seems to act in a catabolic way in chondrocytes and to promote cartilage degradation. To date, there are no reports listed in Pubmed with respect to production of CGRP and its receptors in cartilage. As in bone metabolism where CGRP is described as an anabolic factor by stimulating osteoblast activity and thus bone formation [51,52], one might hypothesize that CGRP has similar anabolic effects in cartilage physiology.

**Table 3:** **Sensory and sympathetic receptors and neurotransmitters expressed in cells and tissues of diathrodial joints**

**Receptors and tissue (cells)**	**Neurotransmitters and tissue (cells)**
**NK1R**	**Substance P**
Chondrocytes of articular cartilage	Chondrocytes of articular cartilage
Chondrocytes of costal cartilage	Chondrocytes of costal cartilage
Proliferating and hypertrophic chondrocytes of the growth plate	Proliferating and hypertrophic chondrocytes of the growth plate
Chondrocytes of OA cartilage	Chondrocytes of OA cartilage
Chondrocytes from fracture callus	Chondrocytes from fracture callus
Osteoblasts	
Osteoclasts and precursors (bone marrow-derived macrophages)	
BMSCs	BMSCs
Synovial fibroblasts	Synovial fibroblasts
	Synovial fluid
**CL receptor/RAMP**	**Calcitonin gene-related peptide**
Osteoblasts	Osteoblasts
Osteoclasts	
BMSCs	BMSCs
	Synovial fluid
**β-Adrenoceptors**	**Tyrosine-hydroxylase (rate-limiting enzyme for catecholamine synthesis)**
Chondrocytes of articular cartilage (β2-AR)	Chondrocytes of the cartilage deep zone
Chondrocytes of costal cartilage (β2-AR)	Costal chondrocytes
Chondrocytes of growth plates (all zones) (β2-AR)	Hypertrophic chondrocytes of the growth plate
Chondrocytes of OA cartilage (β2-AR)	
Chondrogenic progenitor cells from OA cartilage (β2-AR, *in vitro*)	
Osteoblasts (β2-AR)	Osteoblasts
Osteoclasts and precursors (bone marrow-derived macrophages)	Osteoclasts
BMSCs (β2/β3-AR)	Proliferating and chondrogenic/differentiating BMSCs *in vitro*
Synovial cells	Synovial cells during RA pathogenesis
	Synovial fluid
**α1-Adrenoceptors**	
Chondrocytes of OA articular cartilage	
Chondrocytes in fracture callus	
Mesenchymal cells in fracture callus	
**α2-Adrenoceptors**	
Chondrocytes of articular cartilage	
Chondrocytes of the growth plate (different zones)	
Chondrocytes of fracture callus	
Mesenchymal cells of fracture callus	
Osteoblasts	
Osteoclasts and precursors (bone marrow-derived macrophages)	
Synovial cells	
**VIP receptors**	**VIP**
Osteoclasts (VIP-1/-2) and PACAP	Synovial fluid
Osteoblasts (VIP-1/-2)	OA cartilage (?)
Bone marrow-derived macrophages (PACAP)	
Synovial cells (VIP-1/-2)	

AR, adrenoceptor; BMSC, bone marrow-derived stem cell; CL, calcitonin gene-related peptide; NK1R, neurokinin 1 receptor; OA, osteoarthritis; PACAP, pituitary adenylate cyclase-activating polypeptide; RA, rheumatoid arthritis; RAMP, receptor activity-modifying protein; VIP, vasoactive intestinal peptide.

Notably, compared with trauma patients, OA patients have lower VIP concentration in synovial fluid [53]. VIP has been shown to predominantly possess an anti-inflammatory action (reviewed in [54]); it is very efficient at ameliorating the pathology of several models of autoimmune disorders, including RA. Delgado and colleagues [55] showed that treatment with VIP significantly reduced the incidence and severity of collagen-induced arthritis, abrogating joint swelling and the destruction of cartilage and bone. These therapeutic effects were associated with the downregulation of both inflammatory and autoimmune components of the disease [55]. It might be suggested that VIP concentration in synovial fluid is negatively associated with progressive joint damage in OA and has potential as an indicator of disease severity.

Not much is known about production of sympathetic neurotransmitters and their receptors in chondrocytes. β2- and α2a-ARs have been found on growth plate chondrocytes at different developmental stages [56-58], and expression of β2- and α1/α2-ARs was detected in neonatal murine costal chondrocytes [41] and in adult human articular OA chondrocytes [45] (Table 3 and Figure 1). In general, it appears that signaling through β2-ARs interferes with chondrogenic differentiation by inhibiting collagen II, collagen X, and Indian hedgehog expression in part through repression of sox6 and sox9 signaling [56-58]. In addition, we demonstrated that signaling through β2-ARs inhibits apoptosis of murine costal chondrocytes and increases cell-matrix adhesion *in vitro* [41]. In this line, Lai and Mitchell [56] showed that signaling through β2-ARs stimulates murine growth plate chondrocyte proliferation and inhibits terminal differentiation. All together, these sparse data on sympathetic effects in chondrocytes imply that signaling through β-ARs interferes with chondrogenic differentiation by suppressing production of important chondrogenic matrix molecules (Figure 1). This note is corroborated by recent work from Jenei-Lanzl and colleagues [59], who clearly showed that NE stimulation of human adult chondroprogenitor cells inhibits collagen II and glycosaminoglycan production and accelerates the hypertrophic pathway by induction of collagen X and MMP-13 expression, which is opposite to the above reports. In this study, no change in proliferative activity of progenitor cells was observed. This might be because, in the above studies, growth plate chondrocytes were analyzed reflecting an embryonic chondrogenic differentiation status whereas Jenei-Lanzl and colleagues [59] investigated chondrogenic differentiation of adult mesenchymal stem cells and chondrogenic progenitor cells from OA cartilage. In line with this, Li and colleagues [60] showed that β2/3-AR gene and protein expression is increased during adipogenic differentiation of murine bone marrow-derived stem cells (BMSCs). Application of β-AR antagonists positively affects adipogenic differentiation, whereas agonists suppress adipogenic differentiation of BMSCs by downregulating adipogenic marker genes. The authors suggest that these effects are mediated by increased intracellular cAMP level which activates the PKA pathway which presumably mediates downregulation of two key adipogenic transcription factors: C/EBPα and C/EBPβ.

In conclusion, one can speculate that sympathetic neurotransmitters (for example, NE) alter chondrogenic differentiation of chondrogenic progenitor cells by inhibiting chondrocyte hypertrophy via β-ARs during embryonic endochondral ossification (Figure 1 and Table 3). In adults, NE might reduce the self-regeneration capacity of articular cartilage by accelerating the hypertrophic pathway and thus play a role in development and manifestation of OA. In addition, signaling through β-AR inhibits adipogenic differentiation of BMSCs which might be important in the pathogenesis of obesity and osteoporosis in adults.

## Sensory and sympathetic nerve fiber innervation in bone tissues

Several reports have demonstrated an intensive network of sensory and sympathetic nerve fibers within the skeleton, not only in the periosteum but also within trabecular and less in cortical bone, bone marrow, and epiphyseal growth plate [19,23,61-63]. Many of those nerve fibers are associated with blood vessels, but several blood vessel-unrelated nerves and free nerve endings have also been observed. Besides the possibility that sensory and sympathetic nerve fibers have important roles in skeletal pain transmission, accumulating evidence suggests that sensory and sympathetic nerve fibers do have a role in bone remodeling and osteogenic differentiation of precursor cells during skeletal growth. In bone, the areas with the highest metabolic activity receive the richest sensory and sympathetic innervation [64]. This is in line with a study by Offley and colleagues [11], who used selective lesioning of the unmyelinated sensory neural pathway to determine the role of capsaicin-sensitive sensory SP- and CGRP-containing afferents in the maintenance of normal bone balance in skeletally mature rats. Collectively, their results indicate that capsaicin-sensitive sensory neurons contribute to skeletal homeostasis and that lesioning these neurons caused enhanced bone resorption, a reduction in new bone formation, a subsequent loss of trabecular connectivity and thickness, and ultimately an increase in bone fragility [11].

**Figure 2:**
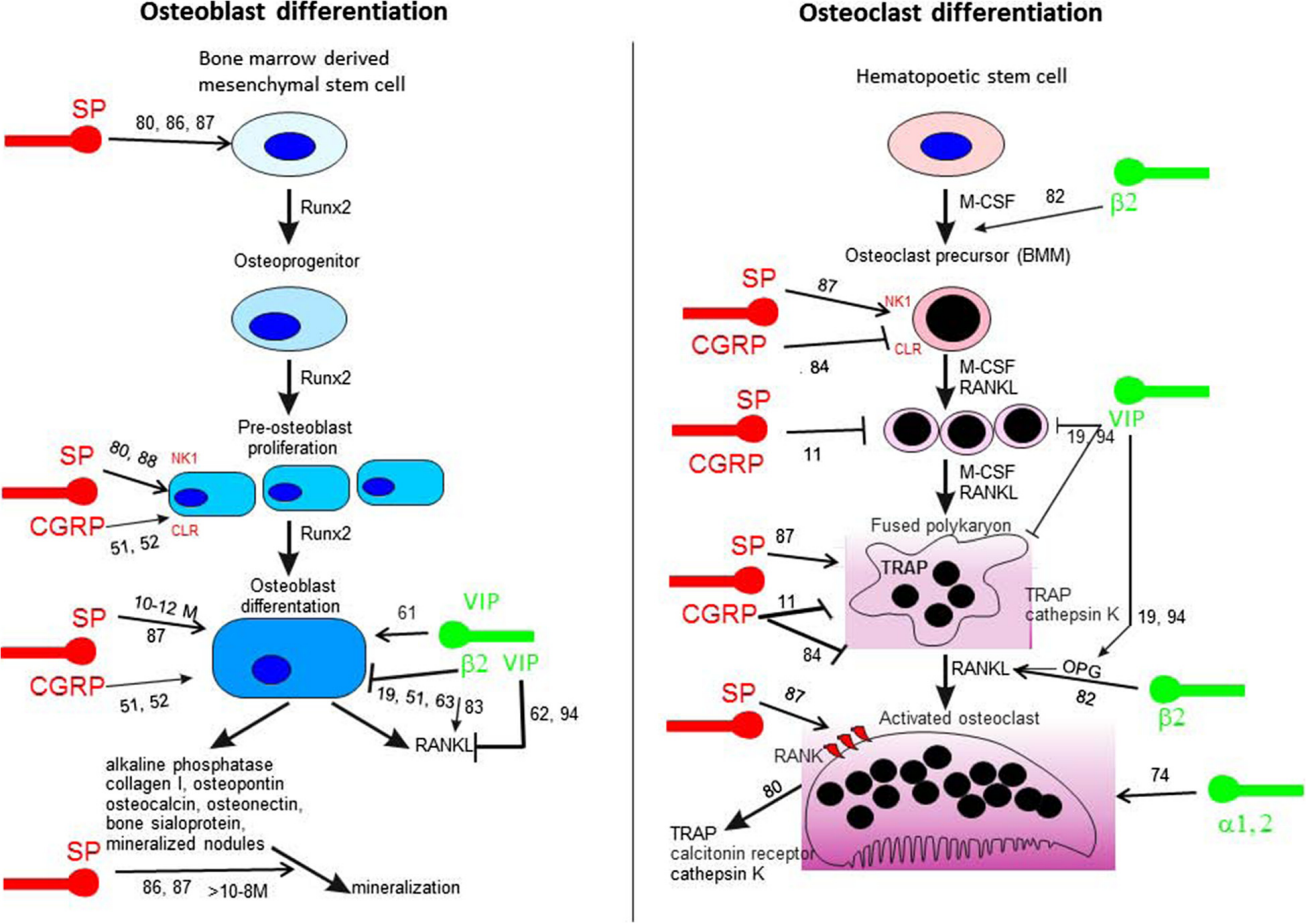
**Neurotransmitters/neuropeptides influence bone homeostasis via their receptors.** Norepinephrine (NE), vasoactive intestinal peptide (VIP), substance P (SP), and calcitonin gene-related peptide (CGRP) affect bone formation and bone resorption by modulating osteogenesis and osteoclastogenesis in different, partly opposing ways. The effects are mediated through neurokinin 1 (NK1) receptor and CGRP receptor (CLR) and both α- and β2-adrenoceptors, depending on catecholamine concentration. A line with an arrow at the end indicates stimulation, and a line with a bar at the end indicates inhibition. The red (green) nerve ending represent sensory (sympathetic) nerve fibers. Numbers indicate references according to bibliography at the end of this review. β2, β2-adrenoceptor; BMM, bone marrow-derived macrophages; M-CSF, granulocyte/macrophage colony-stimulating factor; OPG, osteoprotegerin; RANK, receptor activator of nuclear factor kappa-B; RANKL, receptor activator of nuclear factor kappa-B ligand; TRAP, tartrate-resistant alkaline phosphatase.

Very importantly, bone cells express receptors for many of the neuronal messengers present in these skeletal nerve fibers, and activation of such receptors leads to profound effects on the activity of both osteoblasts and osteoclasts, strongly suggesting the existence of neuro-osteogenic or neuro-immuno-osteogenic interactions (Table 3 and Figure 2).

Experimental studies have provided accumulating evidence that peripheral nerve fibers not only are important in normal bone homeostasis and skeletal growth but also influence repair mechanism after bone trauma (for example, fracture healing). Aro [65] demonstrated that, in denervated limbs, fracture callus size was clearly reduced at a later stage of the healing process. Other studies demonstrated larger callus formation after nerve resection [7,66] and corroborate the observation that bone union is faster and fracture callus volume is increased in patients with head injuries involving coma without clarifying whether this is neuronal, mediated as a direct consequence of the head injury or metabolical, or biochemical as an indirect consequence [1]. At early time points after fracture, TH-, CGRP-, and SP-positive fibers grow into the callus prior to vascularization whereas at later time points these nerve fibers retract to the periosteum [62,67]. Li and colleagues [68] elegantly demonstrated in an angular rat fracture model that innervation of CGRP-positive nerves is higher at the concave compared with the convex site of the fracture. The site-specific changes in CGRP innervation correlate with the amount of bone formation on both the convex and concave sides of angular fractures. Given that the concave side of the angular fracture requires more bone formation than the convex to correct the deformity and that CGRP was clearly more abundant on the concave side, it is reasonable to assume that the peripheral sensory nervous system plays an important role in local bone turnover and that a restored nerve supply could be essential for normal bone regeneration during fracture healing.

VIP has been demonstrated to play an important role in the control of osteoclast formation because destruction of nerves expressing VIP by guanethidine treatment increases the number of osteoclasts [69]. The underlying mechanisms hint to inhibition of osteoclast formation by inhibiting the stimulatory effect of vitamin D3 (1,25(OH)2D3) [70]. This inhibition of osteoclast formation through VIP is mediated via its inhibiting effect on vitamin D3-induced upregulation of receptor activator of nuclear factor kappa-B (RANK) and its ligand (RANKL) and by counteracting a decrease of osteoprotegerin (OPG) caused by vitamin D3 (Figure 2). Thus, VIP would be an excellent candidate that might influence inflammatory processes. Typically, sympathetic nerve fibers carry the two markers TH and NPY, which are important determinants of the catecholaminergic phenotype [71]. However, this can change because there likely exists a bidirectional communication between sympathetic nerve fibers and mesenchymal tissue which influences the neurotransmitter phenotype. For example, periosteal tissue can change the neurotransmitter phenotype of sympathetic nerve fibers [72]. The contact of periosteal cells with sympathetic nerve fibers changes the catecholamine phenotype, leading to a peptidergic and cholinergic phenotype (VIP and acetylcholine instead of NE). It is suggested that several cytokines of the gp130 family and neurotrophic factors determine the switch [73]. During development or under inflammatory conditions such as in arthritis or OA, sympathetic innervation of bone might change, leading to completely different control of bone homeostasis.

An interesting note concerns the involvement of the sympathetic nervous system in the mechanism of bone loss in long-term microgravity in space [74]. The authors suggest that an exposure to prolonged microgravity may enhance sympathetic neural traffic not only to muscle but also to bone. This sympathetic enhancement increases plasma NE level, inhibits osteogenesis, and facilitates bone resorption through β-ARs signaling which in combination leads to reduced bone mass. They suggest that the use of β-AR blockers to prevent bone loss in microgravity may be reasonable, which is in line with suggestions that β-blockers may reduce bone loss and fracture risk in post-menopausal women [12]. On the other hand, Sherman and Chole [75] report that low NE concentrations which are observed after chemical sympathectomy induce bone resorption and decrease bone formation. Therefore, there is not an adequate evidence base to support using β-blockers as a treatment for osteoporosis, nor can β-blockers be regarded as a discriminating risk factor for fracture assessment. Until there are definitive randomized controlled trials with β-blockers, which include fracture as an endpoint, it is unlikely that the current confusing situation will be resolved [12].

Sensory innervation in bone also might play a role in degenerative musculoskeletal disorders as in OA. In diathrodial joints, the subchondral bone contains sensory nerve fibers [76]; however, subchondral regions of normal knee joints are sparsely innervated by SP- and CGRP-nociceptive fibers [77]. SP- and CGRP-positive nerve fibers were identified in the subchondral bone of patients with OA, but it is still unclear whether both sensory nerve fiber types display increased sensitivity or number in OA joints [37]. Both types of nerve fibers have been localized in osteophytes, and perivascular SP-positive nerve fibers were found at the base of osteophytes in horse metacarpophalangeal OA [30,31]. In addition to SP-positive sensory nerve fibers, cells resident in cystic lesions (that is, vascular channels) of the subchondral bone plate of OA knee joints stained positive for SP itself [78]; however, the nature of these cells could not be identified. This note is supported by the observation that SP protein staining in areas of remodeling and in erosion channels of the subchondral bone is increased in OA pathogenesis but no respective nerve fibers were identified [79]. How changes in sensory and sympathetic joint innervation and their respective neurotransmitters contribute to abnormal subchondral bone remodeling and osteophyte formation during the pathogenesis of OA is mostly unknown.

## Sensory neuropeptides and catecholamines and their receptors produced in bone

There is accumulating evidence that sensory and sympathetic neurotransmitters such as SP, αCGRP, VIP, and NE have crucial trophic effects which are essential for proper bone metabolism and bone remodeling. In the recent literature, some comprehensive reviews comment on expression of αCGRP, SP, and sympathetic catecholaminergic (NE) and peptidergic (VIP) neurotransmitters as well as the presence of their receptors on osteocytes, osteoblasts, osteoclasts, bone marrow-derived macrophages, and BMSCs and their contribution to regulation of osteogenic differentiation, osteoclastogenesis, and consequently bone resorption and bone formation in physiological and pathophysiological situations [19,51,54,80-82] (Figure 2 and Table 3).

With respect to the sympathetic nervous system, most cited articles report about the regulation of bone remodeling in adults through alteration in post-synaptic β-AR signaling [51,82]. A recent article by Ma and colleagues [83] addressed the contribution of endogenous sympathetic signaling and NE homeostasis to the control of bone remodeling. They convincingly demonstrated that differentiated osteoblasts, like sympathetic presynaptic neurons, can transport and catabolize NE and thus may contribute to NE clearance within the richly vascularized bone marrow microenvironment. Their findings indicate that the control of NE reuptake by the norepinephrine transporter is an integral part of the homeostatic system whereby bone remodeling is regulated. These data point to the existence of multiple endogenous regulatory pathways modulating bone remodeling via the control of both NE release and NE clearance. Lastly, the authors suggest that drugs blocking norepinephrine transporter activity, which are used for the treatment of depression and attention deficit hyperactivity disorder, may have a deleterious effect on the skeleton.

With respect to sensory neuropeptides, αCGRP-deficient mice suffer from osteopenia [52], and this clearly shows that CGRP can be considered a bone anabolic factor. The osteopenic phenotype was caused by a significant reduction of bone formation; however, osteoblast numbers were not affected, which suggests that CGRP regulates mainly functional activity of osteoblasts and not their mitotic activity. In addition, in mouse bone marrow cultures stimulated to generate osteoclasts by 1,25(OH)2D3, CGRP dose-dependently decreased the numbers of tartrate-resistant alkaline phosphatase-positive multinucleated cells [84]. These data need to be considered together with the notion that osteoporosis is a significant complication of stroke as frequently patients who survive an acute stroke face hip fractures. These stroke-related complications can be attributed to reduced bone mass due to increase in osteoclast-mediated bone resorption and subsequent decreased osteoblast-mediated bone formation (reviewed in [3]).

Notably, a recent study aimed to analyze the effect of CGRP on adipogenic differentiation of BMSCs [85]. The authors demonstrated that simultaneous downregulation of peroxisome proliferator activated receptor-gamma and upregulation of CGRP in rabbit BMSCs efficiently suppressed alcohol-induced adipogenic differentiation while promoting osteogenic differentiation. This is an important observation with respect to development of alcohol-induced osteonecrosis of the femoral head and might have implications for future treatment strategies. Whereas CGRP is *a priori* characterized as a bone anabolic factor [51], SP has both bone resorbing and bone formation activities. Some studies reported opposite effects on bone formation for SP depending on its concentration. Whereas SP concentrations of more than 10^−8^ M stimulate osteoblast differentiation and bone matrix mineralization [86,87], SP concentrations of less than 10^−8^ M block osteogenic differentiation of rat BMSCs but induce proliferation and general protein synthesis [88]. In addition, SP stimulates proliferation of mesenchymal precursor cells and other cells (that is, chondrocytes in a concentration-dependent manner). By blocking the NK1R chemically in rats for 2 weeks, Kingery and colleagues [89] reported a significant reduction in tibial and femoral cancellous bone mineral density as observed in osteoporosis. This strongly suggests a role for SP in maintaining bone integrity and regulation of bone formation and bone resorption. Another observation indicates that ovariectomy which leads to reduced levels of SP at the fracture site was accompanied by impaired mechanical bone properties [90]. These reports indicate a positive effect of SP on bone formation if high concentrations of SP are available and a negative effect if SP concentration is low or if the neuropeptide is absent (Figure 2). In line with these data, our group observed that absence of SP reduces pain sensitivity and mechanical stability of bone in general and negatively impacts on bone structure in an adult murine model of endochondral ossification [67]. The micro-architecture of cortical bone is impaired in SP-deficient mice (*Tachykinin 1* gene-deficient), and the absence of SP reduces bone formation rate indicated by lower bone mass and mineralization. Notably, chemical destruction of the sympathetic nervous system with 6-hydroxy dopamine has even more pronounced effects on bone architecture and bone remodeling by inducing bone resorption without affecting bone formation.

The signaling pathways through which VIP, SP, and CGRP effects on chondro-osteogenic differentiation or osteoclastogenesis are mediated remain largely unknown. Like CGRP, VIP suppresses bone resorptive activities through regulation of RANKL/OPG expression [70]. Yoo and colleagues [91] demonstrated that a decrease in RANKL/OPG ratio was comparable to CGRP/VIP treatment of a pre-osteoblastic cell line either in combination with sheer stress application or separately. They conclude that mechanical stress and VIP/CGRP regulate bone resorptive activities in a similar cellular signal transduction pathway. VIP effects on RANKL and OPG are mediated by an increase in cAMP, indicating that VIP stimulates the PKA/CREB pathway in mouse calvarial osteoblasts, bone marrow, and stromal cells [92,93]. However, RANKL mRNA expression in mouse calvarial osteoblasts can also be mediated by the MEK/ERK pathway [94] and this is in line with other reports demonstrating involvement of MEK/ERK in intracellular signaling by VIP [95]. A recent article [96] presents strong evidence that pro-osteogenic differentiation effects of SP are mediated via activating the Wnt/β-catenin signaling pathway. The same group demonstrates that SP induces proliferation of BMSCs through activation of this pathway [97]. Also, CGRP appears to mediate its effects on osteoblasts through the Wnt/β-catenin pathway [98]. CGRP presumably exerts its anabolic action on bone cells by stimulating canonical Wnt signaling through stabilization of β-catenin and by inhibiting osteoblast apoptosis, thus favoring local bone formation.

All together, these data imply that sensory and sympathetic neurotransmitters have crucial trophic effects which are critical for proper osteogenic differentiation and bone metabolism during embryonic skeletal growth and bone regeneration in adults (that is, after fracture) in addition to their classic neurological actions.

## Conclusions

Sensory and sympathetic nerves and their neurotransmitters are important neuronal effectors regulating bone and cartilage physiology and playing decisive roles in musculoskeletal pathophysiology. Notably, many resident cells of the osteoarticular system contain receptors for sympathetic and sensory neurotransmitters and thus can respond to their stimuli. During endochondral ossification, sensory neuropeptide SP promotes proliferation of stem cells and growth plate chondrocytes, whereas signaling through β-ARs inhibits chondrogenic differentiation of osteo-chondroprogenitor cells and terminal differentiation of chondrocytes. In bone metabolism and bone remodeling, CGRP and VIP have anabolic effects, inducing osteoblast activity and inhibiting osteoclastogenesis, whereas SP also has catabolic effects depending on its concentration. Effects of the sympathetic nervous system on bone homeostasis are complex; however, it is discussed that blocking signaling through β-ARs can prevent bone loss, suggesting that high catecholamine concentrations shift the balance toward bone resorption by inhibiting osteoblast differentiation. During pathogenesis of OA, sympathetic and sensory nerve fibers grow into cartilage from subchondral bone. In late-stage OA synovial tissue, there are more sensory nerves compared with sympathetic nerve fibers; however, it appears that both types of nerves become destroyed during progression of OA. In light of all data, it is more and more evident that sensory and sympathetic nerves and their neurotransmitters critically affect bone and cartilage physiology and are crucially involved in musculoskeletal disorders.

## Note

 This article is part of the series ‘*At the interface between immunology and neurology in rheumatic diseases’*, edited by Rainer Straub. Other articles in this series can be found at http://arthritis-research.com/series/neurology.

## Abbreviations

AR: adrenoceptor
BMSC: bone marrow-derived stem cell
CGRP: calcitonin gene-related peptide
IL: interleukin
MMP: matrix metalloprotease
NE: norepinephrine
NP: neuropilin
NPY: neuropeptide Y
OA: osteoarthritis
OPG: osteoprotegerin
Plx: plexin
RA: rheumatoid arthritis
RANKL: receptor activator of nuclear factor kappa-B ligand
Sema: semaphorin
SP: substance P
TH: tyrosine-hydroxylase
VEGF: vascular endothelial growth factor
VIP: vasoactive intestinal peptide
